# Flux and Passage Enhancement in Hemodialysis by Incorporating Compound Additive into PVDF Polymer Matrix

**DOI:** 10.3390/membranes6040045

**Published:** 2016-10-19

**Authors:** Qinglei Zhang, Xiaolong Lu, Qingzhao Zhang, Lei Zhang, Suoding Li, Shaobin Liu

**Affiliations:** 1 Institute of Biological and Chemical Engineering, Tianjin Polytechnic University, Tianjin 300387, China; bsy_qingleizhang@126.com; 2 Beijing Origin water membrane technology Co. Ltd., Beijing 101400, China; bsy_lisuoding@126.com (S.L.); bsy_liushaobin@126.com (S.L.); 3 State Key Laboratory of Separation Membranes and Membrane Processes, Tianjin 300387, China; 4 Xintai Traditional Chinese Medical Hospital, Xintai 271200, China; 5 Xintai Second Peoples Hospital, Xintai 271219, China; xtseytw@163.com

**Keywords:** PVDF, PVP, PEG, hemodialysis membrane, structure, performance

## Abstract

In this study, Polyvinylidene fluoride (PVDF) hollow fiber hemodialysis membranes were prepared by non-solvent induced phase separation (NIPS) with compound addtive. The compound additive was made with polyvinyl pyrrolidone (PVP) and Poly ethylene glycol (PEG). The results showed that the modified PVDF membrane had better separation performance than virgin PVDF membrane. The UF flux of modified PVDF membrane can reach 684 L·h^−1^·m^−2^ and lysozyme (LZM) passage is 72.6% while virgin PVDF membrane is 313 L·h^−1^·m^−2^ and 53.2%. At the same time, the biocompatibility of PVDF membranes was also improved. Compared with commercial polysulfone hemodialysis membrane (Fresenius F60S membrane), the modified PVDF membrane had better mechanical and separation performance. The stress and tensile elongation of modified PVDF membrane was 0.94 MPa and 352% while Fresenius F60S membrane was 0.79 MPa and 59%. The LZM passage reached 72.6% while Fresenius F60S membrane was 54.4%. It was proven that the modified PVDF membrane showed better hydrophilicity, antithrombogenicity, less BSA adsorption, and lower hemolytic ratio and adhesion of platelets. Water contact angle and BSA adsorption of the modified PVDF membrane are 38° and 45 mg/m^2^ while Fresenius F60S membrane are 64° and 235 mg/m^2^. Prothrombin time (PT) and activated partial thromboplastin time (APTT) of the modified PVDF membrane are 56.5 s and 25.8 s while Fresenius F60S membrane is 35.7 s and 16.6 s. However, further biocompatibility evaluation is needed to obtain a more comprehensive conclusion.

## 1. Introduction

Hemodialysis (HD) is a relatively safe purification technique, which partly replaces renal function. Excess moisture and metabolic wastes were removed by HD, and in the meantime, calcium ion, bicarbonate ion, and other substances can be supplied. The core element is Ultrafiltration membrane [1]. The core aim for hemodialysis is to remove “middle” and “small” molecules toxin, such as β2-Microglobulin (β2-MG) and urea nitrogen. Clearance and Ultrafiltration coefficient are two main parameters of the dialyzer. Cellulose membranes are widely used for hemodialysis because of their hydrogel structure and small thickness, which provide a very effective removal for small solutes such as urea and creatinine. However, these membranes provide relatively little clearance for “middle” molecules and cause complement activation upon contacting with blood. For dialysis membrane, the polymer materials should have excellent biocompatibility, fiber spinning ability, and appropriate morphology structure. Nowadays, polyethersulfone (PES) and polysulfone (PS) hemodialysis membranes showed better biocompatibility, functional effectiveness, and small-molecular substances clearance. So they are widely used in hemodialysis [2,3,4,5,6,7]. However, biocompatibility and clear efficiency for “middle” molecules toxin of these materials were not ideal enough. For example, anticoagulants (such as hirudin or heparin) should be added during hemodialysis owing to the poor anticoagulation property of commercial membranes. Studies on developing high performance hemodialysis membranes obtained worldwide attention and many works have been focused on the modification of current membranes for the purpose of enhancing the hemodialysis properties. Though modification of current materials is an effective method to improve the biocompatibility of hemodialysis membranes, it is far from application owing to the complexity of the chemical and other modification process. The discovery of new materials to prepare membranes with better biocompatibility and hemodialysis properties is urgently needed.

Polyvinylidene fluoride (PVDF), a widely used material in the field of water purification, which has recently received great attention as a membrane material with regard to its outstanding properties, such as high mechanical strength, thermal stability, anti-ultraviolet radiation, smooth surface, and low protein adsorption, compared with other polymeric materials [8,9,10,11,12]. Laroche G et al. [13] pointed out that PVDF had excellent biocompatibility and minimal cell protein adsorption. Polyvinylpyrrolidone (PVP) was widely used in the biomedical field, due to its excellent biocompatibility. Studies [14,15,16] had shown that the PS dialysis membrane blended with Polyvinylpyrrolidone (PVP) has excellent biocompatibility.

The previous results of our research showed that the PVDF membranes were prepared by blended with Poly ethylene glycol (PEG) polymers, which had good mechanical performance and biocompatibility, but worse Ultrafiltration (UF) flux of pure water and antithrombogenicity [17,18]. In this study, an attempt was made to improve separation performance of membrane, especially UF flux by exploring the effects of PVP molecular weight and content on the membrane structure and performance.

## 2. Materials and Methods

### 2.1. Materials

PVDF (1010, SOLVAY, Lyon, France); Polyvinylpyrrolidone (PVP), Poly ethylene glycol (PEG) (Sigma-Aldrich Trading Co., Ltd. Shanghai, China); Fresenius F60S membranes (Fresenius Medical Care AG, Frankfurter, France); Lysozyme and urea (Beijing Pubo Xin Biological Technology Co., Ltd. Beijing, China); Albumin from bovine serum (BSA) (Shanghai biomedical engineering technical service company, Shanghai, China); *N*,*N*-dimethylacetamide (DMAc) (Samsung company, Seoul, Korea). PT and APTT were purchased from Wuhan Sailisi biomedical technical company, China; All the reagents used in the study were of reagent grade.

### 2.2. Preparations of PVDF Membranes

The PVDF hollow fiber membranes were prepared by NIPS. Casting dopes of virgin PVDF were prepared by adding PEG while modified PVDF membranes were prepared by blending with PEG and PVP into the solvent, including 1,4-diethylene dioxide (5.2 wt %) and DMAc (58 wt %), followed by stirring at 70 °C until the solution became homogeneous. PVP, 1,4-diethylene dioxide and PEG worked as pore-forming agents to produce porous structures in the membrane. The dope solution was then transferred into a tank and kept at a constant temperature of 70 °C for 12 h to eliminate the air bubbles before being used. The casting solution and bore fluid were passed through the orifice and inner tube, respectively. The nascent membranes were taken up at a drawing rate of 70 m·min^−1^ and immersed in pure water for at least 48 h to remove the residual DMAc, then kept in glycerol aqueous solution. Finally, the membranes were dried in ambient air. The inner diameter and wall thicknesses of PVDF hollow fiber membranes were 200 and 40 μm, respectively. The spinning parameters were summarized in Table 1 for quick reference. The flat membranes were prepared by the next step. After air bubbles were removed completely, the dope solution was spread onto a plate by a glass blade, and different membranes were obtained by evaporating the dope solution at 30 °C temperature. The membranes were removed from plate by immersing into 30 °C water bath, and kept in an oven with temperature of 60 °C for 8 h to remove the possible solvent residues.

### 2.3. Morphology and Maximum Pore Size

Morphology studies of PVDF HFMs were carried out using a scanning electron microscope (Hitachi S-4800, HITACHI, Tokyo, Japan). The membranes were immersed in liquid nitrogen and fractured carefully. The specimens were put on a metal support and dried under vacuum. Then, the specimen was coated by sputtering gold under vacuum using a Bal-Tec SCD 005 sputter coater (HITACHI, Tokyo, Japan). Samples were observed under an electron microscope at 10 kV.

The membranes were soaked in ethanol for 15 min. At room temperature, the membrane was immersed in ethanol, and then nitrogen can be pressurized into the inside. The bubble point pressure, *P*, reached when the first string of bubbles came from the walls of the membrane. The maximum pore size can be calculated according to Equation (1) [19]:

(1) $$r=\;\frac{0.06378}{2P}$$

where *r* is the pore radius (μm), *P* is bubble point pressure (MPa), and the ethanol surface tension is 22.3 mN/m.

The membrane porosity, ε, was measured by soaking the membrane in pure water for 2 h, and then the membrane surface was dried by filter paper. The membrane was weighed before and after absorption of pure water. The porosity was calculated using Equation (2):

(2) $$\varepsilon =\frac{\left(W_{w}-W_{d}\right)/\rho_{W}}{\left(W_{w}-W_{d}\right)/\rho_{W}+W_{d}/\rho_{p}}\times 100\%$$

where ε is the porosity of the membrane (%), *W_w_* is the mass of the wet membrane, *W_d_* is the mass of the dry membrane, ρ*_w_* is the density of pure water (1.0 g/cm^3^), and ρ*_p_* is the density of the membrane (1.78 g/cm^3^).

### 2.4. Mechanical Performance

The mechanical performance of the membranes was measured using an electronic single yarn strength tester (YG061 F/PC, Lanzhou Electron Instrument Co., Ltd., Lanzhou, China) at room temperature. Each sample was clamped at both ends and unidirectional stretched at a constant elongation rate of 500 mm/min with an initial length of 10 cm. Specimens were selected randomly and tested from each batch of the dried hollow fiber sample. The tensile elongation and tensile stress at break were determined. The experiments were be repeated for five times to get the average data.

### 2.5. UF Flux of Pure Water

Self-assembly widgets were made with 20 pieces of PVDF hollow fiber membranes by epoxy resin cast. The inner diameter and wall thicknesses of PVDF membranes were 200 and 40 μm, respectively. The length of membranes was about 20 cm. The surface area of membranes was 25 cm^2^. Ultrafiltration flux was measured by internal pressure method with the feed rate of pure water was 200 mL/min. The membranes were preloaded under 0.2 MPa for about 20 min. After adjusting the test temperature (25 °C), The UF flux was measured with the inlet pressure (0.102 MPa) and the outlet pressure (0.098 MPa). The transmembrane pressure (TMP) was 0.1 MPa.

The UF flux was calculated using Equation (3):

(3) $$J=\frac{V}{S\cdot t}$$

where *J* is the UF flux of pure water (L·h^−1^·m^−2^), *V* is the volume of the permeate flow (L), *S* is effective membrane area (m^2^) and *t* is sampling time (h).

### 2.6. Rejection and Passage

To easily go on study, β_2_-Microglobulin (β_2_-MG) and human serum albumin were replaced by lysozyme (LZM) and Albumin from bovine serum (BSA). LZM and β_2_-MG have the similar molecular weight as LZM is 14 KDa while β_2_-MG is 11.8 KDa. The molecules of them are both positive charge and globulin. At the same time, isoelectric point of them is relatively similar (LZM was 4.6 and β_2_-MG 5.7). The molecular weight of BSA is 67 KDa while human serum albumin is 69 KDa. BSA rejection, Urea, and LZM passage were measured in the same method as UF flux. After preflush, the membranes with the solute for about 30 min under temperature (25 °C), The BSA rejection, Urea, and LZM passage of membranes were measured at the inlet pressure (0.100 MPa) and outlet pressure (0.060 MPa). The initial concentration of urea, LZM and BSA were 2000, 35 and 1000 mg/L, respectively.

The rejection of BSA(*R*) was calculated by the following Equation (4):

(4) $$R=1-\frac{Cp}{C_{r}}$$

where *C_p_* and *C_r_* (mg·L^−1^) are BSA concentrations of permeate and remaining solution.

The urea and LZM passage was calculated by the following Equation (5):

(5) $$P=\frac{C_{p}}{C_{r}}$$

where *C_p_* and *C_r_* (mg·L^−1^) are the concentrations of permeate and remaining solution. The concentration of different solute is determined by UV-Vis spectrophotometer.

### 2.7. Hydrophilic Test

The hydrophilicity of membrane surface was characterized on the basis of water contact angle measurement by YH-168A type contact angle goniometer. The membranes (2 cm × 2 cm) rinsed with ethanol and then put in 60 °C ovens drying for 2 h. The contact angle was measured 10 s after water was dropped on the airside surface of membranes. At the same time, we use the airside surface as feed side. At least eight measurements were averaged to get a reliable value.

### 2.8. Protein Adsorption

The protein adsorption experiments were carried out with bovine serum albumin (BSA), which were dissolved in the phosphate-buffered saline solution (PBS, pH 7.4) with a concentration of 1 mg·mL^−1^. The membranes with an area of 10 cm × 10 cm were incubated in PBS solution for 24 h and then immersed in the protein solution for 12 h at 25 °C. The BSA concentrations before and after membrane adsorption were measured by UV-Vis spectrophotometer.

#### 2.8.1. Hemolytic Ratio (HR)

A sample of 4 mL fresh venous blood was collected from a healthy rabbit using centrifugal tubes. The blood was mixed with heparin sodium as an anticoagulant. The membranes were put into centrifuge tubes followed by the addition of 1000 µL 0.9% physiological saline. They were preheated for 30 min in a shaking bath. Then 20 µL diluted whole blood was dropped into the centrifuge tubes. The tubes were soaked in water-bath for 60 min at 37 °C. Triple-distilled water and 0.9% physiological saline were used as positive and negative controls, respectively. Then they were treated in the same way as above during this work. 200 µL of the upper clear solution was pipetted to 96-well plates. The absorbency was measured by THERMO Varioskan Flash 3001type (Bao Cheng Biological Technology Co., Ltd. Hangzhou, China); full wavelength multifunctional enzyme mark instrument at 545 nm. The hemolytic ratio (HR) was calculated as following Equation (6):

(6) $$HR=\frac{AS-AN}{AP-AN}$$

where *AS*, *AN*, *AP* are the absorbency of sample, negative control, and positive control.

#### 2.8.2. Blood Coagulation Time

Blood coagulation time was characterized from extrinsic and intrinsic pathway of blood coagulation by prothrombin time (PT) and activated partial thromboplastin time (APTT). A sample of 20 mL fresh heart blood was collected from a healthy rabbit using centrifugal tubes. The blood was mixed with sodium citrate as an anticoagulant (anticoagulant to blood ratio, 1:9 *v*/*v*), and fresh plasma was obtained after centrifuging at 2000 g for 15 min at 4 °C. The PT test method was described as follows: Firstly, all the samples (2.0 cm × 0.6 cm) were put into the magnetic cup. Secondly, 50 μL plasma was put into the magnetic cup, and incubated for 3 min at 37 °C. The reaction was initiated with 100 μL of thromboplastin. Then the PT was measured by CA-50 type automated blood coagulation analyzer (Sysmex Corporation, Kobe, Japan). The APTT test method was described as follows: Firstly, all the samples were put into the magnetic cup. Then 50 μL plasma and 50 μL APTT mixture was put into the magnetic cup, and incubated for 3 min at 37 °C. Afterward, the reaction was initiated with 50 μL CaCl_2_). Then the APTT was measured by CA-50 type automated blood coagulation analyzer.

#### 2.8.3. Platelets Adhesion

A sample of 20 mL fresh heart blood was collected from a healthy rabbit using centrifugal tubes. Before the experiment, all the appliances, reagents, and membranes (0.5 cm × 0.5 cm) were sterilized by 75% alcohol for 15 min, and then the membranes were immersed into isotonic phosphate buffer solution (PBS, pH 7.4) for 30 min. Then the membranes were placed in 48-well plates. 500 µL of PRP was dropped into each well and then it was incubated with the membrane for 90 min at 37 °C. The dehydration was carried out through a series of ethanol/PBS mixtures with increasing ethanol concentrations. After vacuum-drying, the platelet-attached membranes were coated with a gold layer and the FESEM images were recorded by QuanTa 200 type field emission scanning electron microscopy (Beijing Perlong Medical Co., Ltd. Beijing, China) at the magnification of 1500× and 5000×.

## 3. Results

### 3.1. Effect of PVP Molecular Weight on PVDF Membranes Structure and Performance

The PVDF membranes were prepared by non-solvent-induced phase separation (NIPS). A series of modified PVDF membranes were prepared by blended with PEG and PVP. The PVDF content was 22 wt %. PEG content was 15.8 wt % and PVP content was 3 wt %. The cross-sectional SEM micrograph of virgin PVDF membrane (PEG 18.8 wt %) is M-0. The cross-sectional SEM micrographs of the modified PVDF membranes are M-a, M-b, and M-c with PVP molecular weights 6 KDa, 10 KDa, and 30 KDa, respectively.

**Figure 1:**
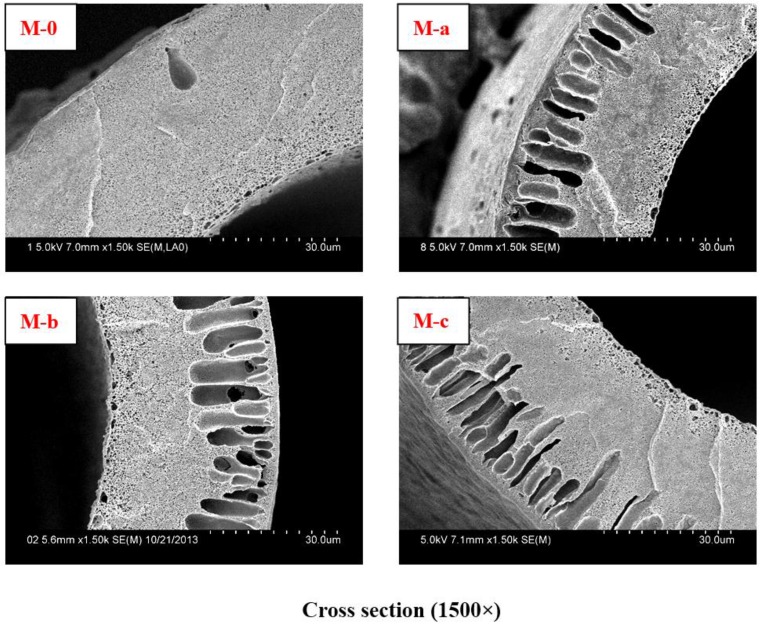
Cross-sectional SEM micrographs with different PVP molecular weights in membranes. The cross-sectional SEM micrograph of Virgin PVDF membrane is M-0. The cross-sectional SEM micrographs of modified PVDF membranes are M-a, M-b, and M-c with PVP molecular weights 6 KDa, 10 KDa, and 30 KDa, respectively.

The cross-sectional SEM morphologies of different PVDF membranes are shown in Figure 1. The modified PVDF membranes exhibited typical asymmetric structure with a skin layer on outer, an intermediate layer with finger-like structure, and a bottom layer of fully developed macrospores. From Figure 1, it can be seen that the finger-like structure becomes more obvious.

**Figure 2:**
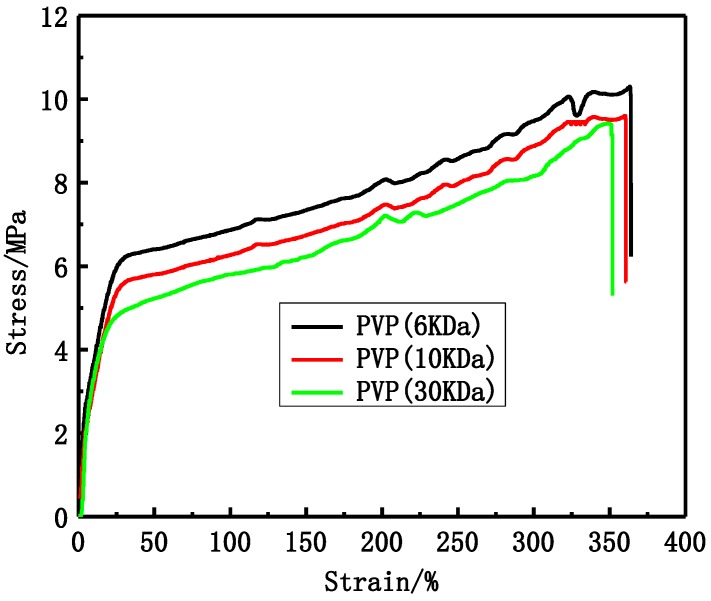
The stress-strain curves of different PVP molecular weight PVDF membranes.

The tensile stress and bursting pressure at break decrease with increasing PVP molecular weight, as shown in Figure 2 and Table 2. The tensile stress at break decreases from 10.3 MPa to 9.4 MPa and the bursting pressure decreases from 0.595 MPa to 0.495 MPa.

The BSA rejection, LZM and urea passage of different modified PVDF membranes are shown in Table 3. From the results, it can be seen that the rejection of BSA decreases while the passage of LZM increases with increasing PVP molecular weight.

As shown in Table 3, The UF flux of pure water increases from 483 L·h^−1^·m^−2^ to 684 L·h^−1^·m^−2^ and the passage of LZM increases from 52.5% to 72.6% with PVP molecular weight increasing from 6 to 30 KDa. UF flux was measured with the inlet pressure (0.102 MPa) and the outlet pressure (0.098 MPa). The water passed through the tube side.

### 3.2. Effect of PVP Content on Membrane Structure and Performance

A series of modified PVDF membranes were prepared by blended with PEG and PVP (30 KDa). The PVDF content was 22.0 wt %, and the total content of PEG and PVP was 18.8 wt %. The SEM micrographs of modified PVDF membranes are M-1, M-3, and M-5 with PVP content 1.0, 3.0, and 5.0 wt %, respectively.

**Figure 3:**
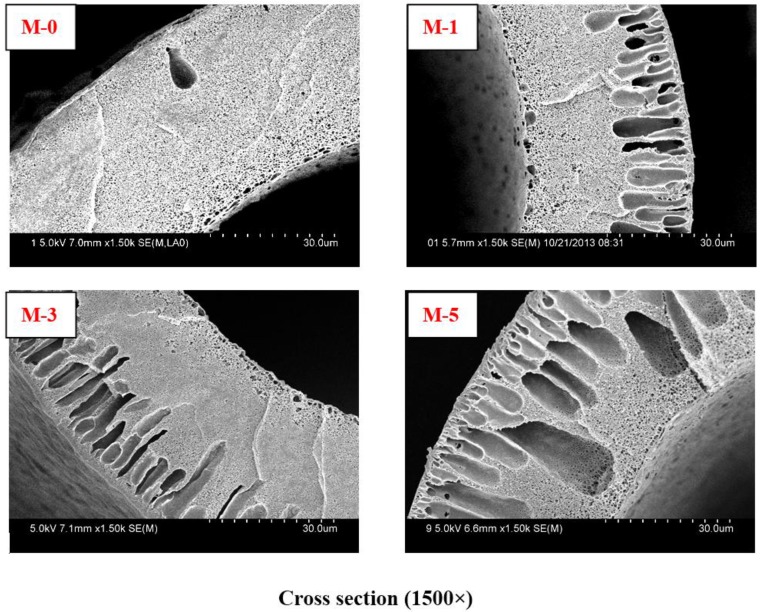
Cross-sectional SEM micrographs with different PVP content in membranes. The cross-sectional SEM micrograph of virgin PVDF membrane is M-0. The cross-sectional SEM micrographs of modified PVDF membranes are M-1, M-3, and M-5 with PVP content 1.0%, 3.0%, and 5.0 wt %, respectively.

Figure 3 shows the cross-sectional SEM morphologies of different PVP content PVDF hollow fiber membranes. The cross-sectional structures of modified PVDF membranes are different. Long finger-like pores are present near the outer walls of the hollow fiber membranes, while sponge-like structures are possessed by the center of the hollow fiber membranes and the inner walls.

**Figure 4:**
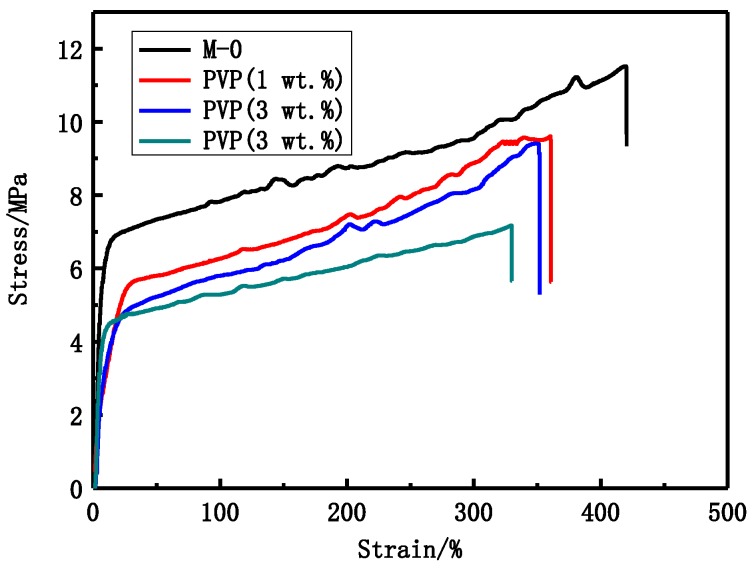
The stress-strain curves of different PVP content PVDF membranes.

As shown in Figure 4 and Table 4, the tensile stress and bursting pressure at break decrease with increasing PVP content. The tensile stress decreases from 9.6 MPa to 7.2 MPa and the bursting pressure decreases from 0.565 MPa to 0.370 MPa while the porosity increases from 84.1% to 90.4% with increasing PVP content. As can be seen from the Figure 3, the proportion of the finger-like structure becomes larger and larger with increasing PVP content.

UF flux of pure water, BSA rejection, LZM, and urea passage of PVDF hollow fiber membranes prepared with different PVP content are shown in Table 5. With PVP content increasing from 1 wt % to 5 wt %, BSA rejection rate decreases from 92.4% to 88.2% while LZM passage increases from 58.9% to 82.5%. The UF flux increases from 512 L·h^−1^·m^−2^ to 842 L·h^−1^·m^−2^.

### 3.3. Contrastive Study of PVDF and Commercial Fresenius F60S Membrane

From the results, it can be known that the passage of lysozyme and UF flux were effectively improved. When PVP molecular weight is 30 KDa and the content is 3.0 wt %, the modified PVDF membrane has better mechanical and separation performance. The modified PVDF membrane (M-c/M-3) was chosen to research the biocompatibility. M-0 and M-c/M-3 are the virgin and modified PVDF membranes.

#### 3.3.1. Morphology and Structure

**Figure 5:**
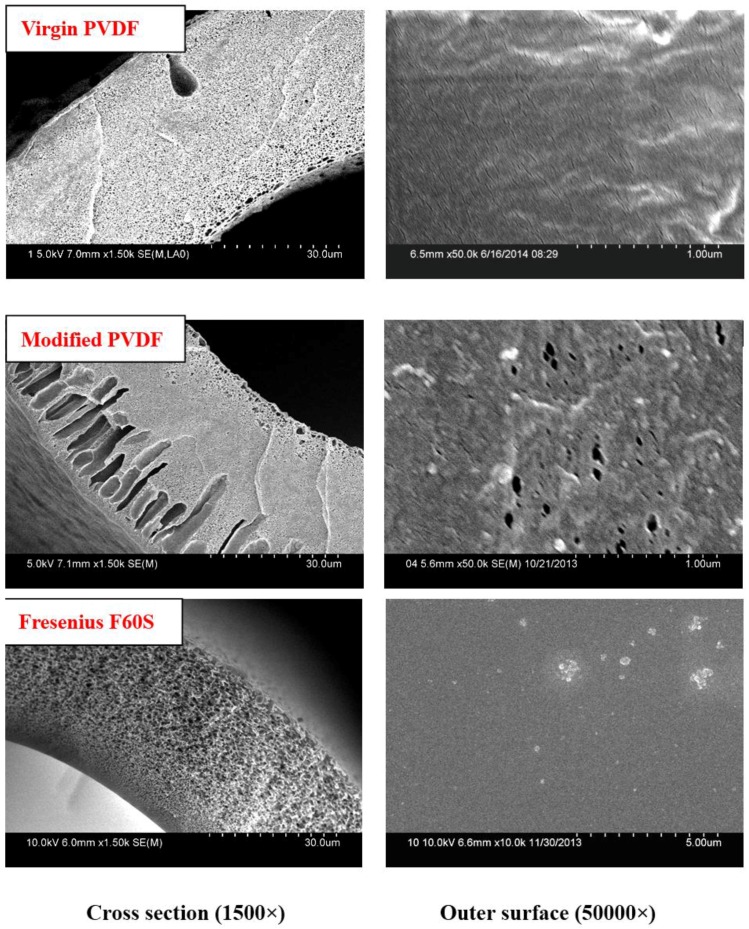
Cross-sectional and outer surface SEM micrographs of PVDF and Fresenius F60S membranes. M-0 and M-3/M-c are the Virgin and modified PVDF membranes.

The Cross-section and outer surface SEM micrographs of PVDF and Fresenius F60S membrane are shown in Figure 5. There are no finger-like pores in virgin PVDF and Fresenius F60S membranes. The virgin PVDF membrane is M-0 without PVP. The modified PVDF membrane exhibits more finger-like pores than PVDF membrane.

#### 3.3.2. Mechanical and Separation Properties

**Figure 6:**
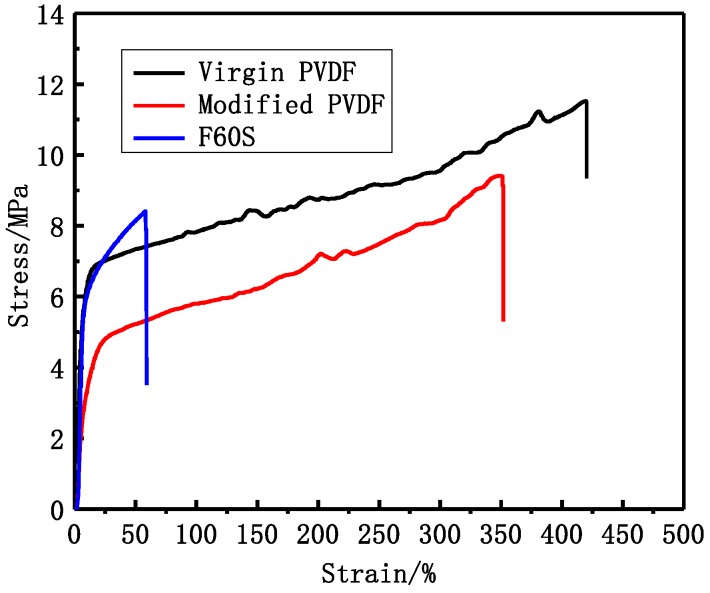
The PVDF and Fresenius F60S membranes stress-strain curve.

From Figure 6 and Table 6, it can be seen that virgin and modified PVDF membranes have better mechanical performance than Fresenius F60S membrane. Mechanical performance of modified PVDF membrane is reduced by a certain degree. The modified PVDF membrane exhibits more finger-like pores than virgin PVDF membrane. However, the mechanical performance of modified PVDF membrane can reach the requirement of hemodialysis. The tensile stress and tensile elongation of modified PVDF membrane are 9.4 MPa and 352% while Fresenius F60S membrane is 7.9 MPa and 59%.

From Table 7, it can be known that the UF flux and LZM passage of modified PVDF membrane were effectively improved. The UF flux of modified PVDF membrane can reach 684 L·h^−1^·m^−2^ and LZM passage is 72.6% while PVDF membrane is 313 L·h^−1^·m^−2^ and 53.2%. The UF flux of Fresenius F60S membrane was 217 L·h^−1^·m^−2^.

#### 3.3.3. Water Contact Angle and BSA Adsorption

From Table 8, it can be seen that the virgin PVDF and modified PVDF membranes have better hydrophilicity and less BSA adsorption than Fresenius F60S membrane. Water contact angle and BSA adsorption of modified PVDF membrane were 38° and 45 mg/m^2^ while Fresenius F60S membrane were 64° and 235 mg/m^2^.

#### 3.3.4. Hemolysis Ratio

Hemolytic ratio experiment is one of the general and important methods to appraise blood compatibility. Table 9 exhibits that hemolytic rate of PVDF and modified PVDF membranes are lower than Fresenius F60S membrane no matter at which sample, indicating that the red blood cell (RBC) broken ratio of PVDF material is less than PSF material. The modified PVDF membrane has the lowest hemolytic ratio, which is about 0.3%. The hemolytic ratio of both PVDF and PSF membranes were less than 5%.

#### 3.3.5. Blood Coagulation Time

**Figure 7:**
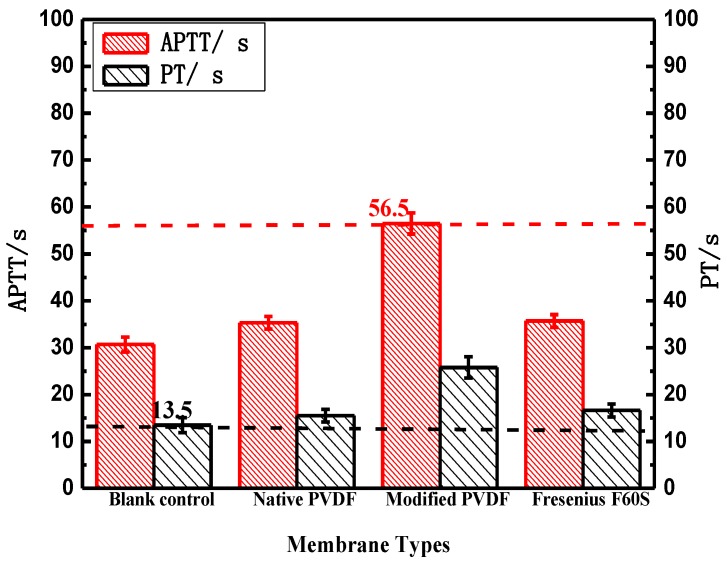
The blood coagulation time of PVDF and Fresenius F60S membranes. * *p* < 0.05, when PVDF membranes compared with blank control.

As illustrated in Figure 7, PT of both PVDF and Fresenius F60S membranes are approximate to blank control while the modified PVDF membrane is longer than blank control. PT of the modified PVDF membrane is 9.2 s longer than Fresenius F60S membrane. That can be explained by the high hydrophilicity surface of the modified PVDF membrane. The modified PVDF has higher hydrophilicity (38°) by blending with PVP than virgin PVDF (52°) and F60S (64°) membranes. PVP with high hydrophilic and flexibility can combine in thrombin, which prevents fibrinogen from changing into fibrin. APTT of both PVDF and Fresenius F60S membranes are longer than blank control, indicating both materials have intrinsic antithrombogenicity. APTT of the modified PVDF membrane is 20.7 s longer than Fresenius F60S membrane.

#### 3.3.6. Platelet Adsorption

For blood-contacting materials, adhesion of platelets onto surfaces of materials is a key event in thrombus formation. Protein adsorb on polymer material surface on the first occurrence when it contacts with blood, especially fibrinogen, and causes platelet activation. Then the activated platelets accelerate thrombosis as they promote thrombin formation and platelet aggregation. Thus, platelet adhesion is another important parameter to evaluate the blood compatibility.

**Figure 8:**
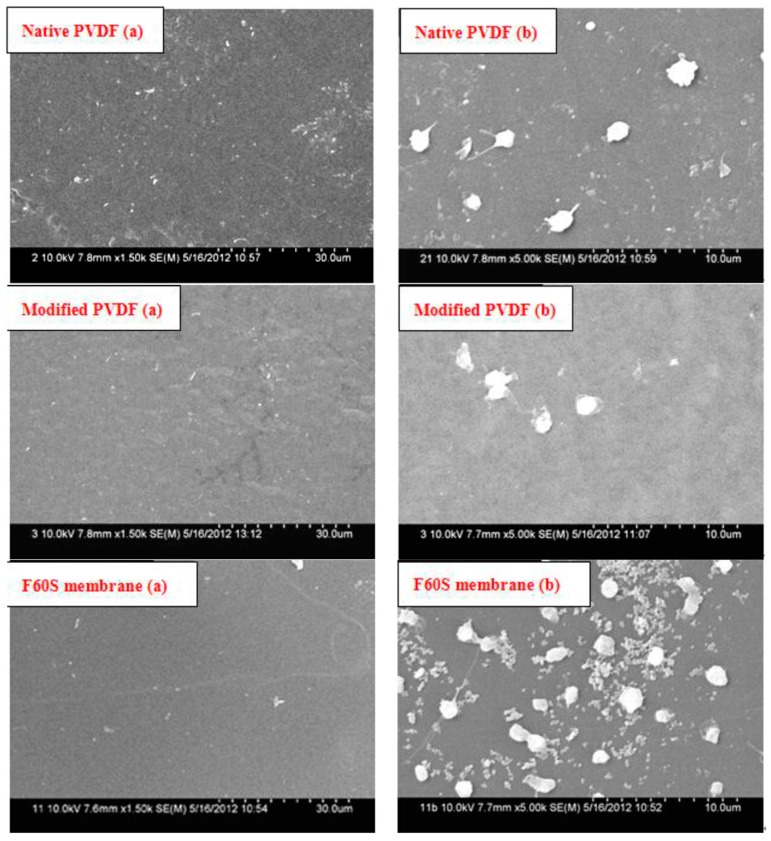
Platelet adsorption of PVDF and Fresenius F60S membranes. (**a**) Original membrane surface feature 1500× (**b**) Membrane surface feature after platelet adhesion 5000×.

The diversity among different membrane surfaces for platelet adhesion, activation, aggregation, and amount were investigated by FESEM, as seen in Figure 8. The shapes of platelets of PVDF and Fresenius F60S membranes express rounded morphology; nearly no pseudopodium and deformation are found membranes, which indicate that both PVDF and Fresenius F60S membranes will not activate platelets. In addition, the platelet adhesion amount of the modified PVDF membrane was much lower than that of PVDF and Fresenius F60S membranes. It revealed that the anti-platelet adhesion of the modified PVDF membrane was better than PVDF and Fresenius F60S membranes.

## 4. Discussions

### 4.1. PVP Effects on Membranes Structure and Performance

In this study, an attempt was made to improve separation performance of membrane by exploring the effects of PVP molecular weight and content on membrane structure and performance to get a better modified PVDF membrane. At the same time, we did some research on the biocompatibility of PVDF and Fresenius F60S membranes.

PVP molecular weight has an important influence on PVDF membranes structure and performance. The modified PVDF membranes exhibited typical asymmetric structure (Figure 1), which becomes more obvious with increasing PVP molecular weight. There are two main reasons accounting for that. One is due to the difference of PVP molecular weight. Higher molecular weight PVP is more conducive to form larger pores than low molecular weight PVP. The other is due to the casting solution viscosity, which can affect the solvent and non-solvent diffusion rate. Higher molecular weight PVP can increase casting solution viscosity. The tensile stress and bursting pressure at break (Figure 2 and Table 2) decrease with increasing PVP molecular weight. That can be explained by cross-sectional structures of membranes. With PVP molecular weight increasing, the finger-like pores and porosity become larger and larger, which can decrease the membrane mechanical performance. The rejection of BSA decreases while the passage of LZM increases (Table 3) with increasing PVP molecular weight. The reason is that the maximum pore size can affect the BSA rejection and LZM passage. The urea passages of different modified PVDF membranes are similar. That can be explained by the smaller molecular weight of urea, which can easily go through membrane. The UF flux of pure water increases with PVP molecular weight increasing from 6 to 30 KDa. Low molecular weight additives have higher solubility than high molecular weight additives and therefore can be washed out together with the solvent from membranes to the coagulation bath. However, the higher molecular weight additives are difficult to flow from membranes. There are more additives staying in membranes. PVP is a hydrophilicity polymer, which can increase the hydrophilicity of PVDF membranes. At the same time, the maximum pore size becomes larger and larger with increasing PVP molecular weight, which can also increase the UF flux.

PVP content also has an important influence on the PVDF membranes structure and performance. The modified PVDF membranes have typical asymmetric structure that was formed via NIPS. The structure can be due to the rapid precipitation resulting in finger-like pores and the slow precipitation giving the sponge-like structure. From Figure 3, it can be seen that the asymmetric structure become more obvious with increasing PVP content. The casting solution viscosity becomes larger and larger with increasing PVP content. The viscosity can affect the solvent and non-solvent diffusion rate. As shown in Table 4 and Figure 4, the tensile stress and bursting pressure at break decrease with increasing PVP content. The results indicate that the porosity can affect mechanical performance of membranes. BSA rejection rate decreases while LZM passage (Table 5) increases with increasing PVP content. That can be explained by the maximum pore size that increases with increasing PVP content. The increase of UF flux may be attributed to the increasing pore size and the hydrophilicity of modified PVDF membranes.

### 4.2. Structure and Performance of PVDF and Commercial Fresenius F60S Membrane

The virgin and modified PVDF membranes have better mechanical performance than Fresenius F60S membrane. The PVDF membranes and Fresenius F60S membrane have different mechanical performance, which mainly depends on the different materials and different cross-sectional structures (Figure 5). The modified PVDF membrane exhibits more finger-like pores than PVDF membrane. There are two reasons for that. One is that PVP and PEG have different compatibility with PVDF polymer (δ PVDF = 21.7, δ PEG6000 = 22.4 [20], δ PVP = 32.5 [21]). PEG has a better compatibility with PVDF than PVP. Second is the different molecular weight between PEG and PVP. High molecular weight additive is more conducive to forming larger pores. The modified PVDF membrane has higher UF flux and LZM passage than virgin PVDF membrane. That can be explained by the different molecular weight of additives. PEG (6 KDa) has higher solubility than high molecular weight PVP (30 KDa) and, therefore, it can be washed out together with the solvent from membranes to the coagulation bath. However, the higher molecular weight additives are difficult to flow from membranes. There are more additives staying in membranes. PVP is a hydrophilicity polymer, which can increase the hydrophilicity of modified PVDF membrane (38°). At the same time, there are more open pores on the surface of modified PVDF membrane, which can also increase the UF flux and LZM passage. Compared with Fresenius F60S membrane, the modified PVDF membrane has higher UF flux and LZM passage while worse rejection of BSA than Fresenius F60S membrane. The reason for that can be explained by the appropriate morphology and the higher hydrophilicity of modified PVDF membrane.

The modified PVDF membrane showed the better antithrombogenicity, hydrophilicity, less BSA adsorption, and lower hemolytic ratio and adhesion of platelets than virgin PVDF and F60S membranes (Table 8 and Table 9 and Figure 8). Water contact angle is a convenient way to assess the wet ability properties of membrane surface and provides information on the interaction energy between the surface and the liquid. When membrane is used for blood separation, protein adsorption is the first stage of the interactions of membrane and blood, which may lead to further undesirable results. PVDF membranes have better hydrophilicity and less BSA adsorption than Fresenius F60S membrane. This is because the M-3 bond energy of PVDF is high, and the fluorine atom sizes are small and tightly packed, which makes the fluorine carbon chain oleophobic effect stronger than hydrocarbon chain. PEG and PVP is polymer with hydrophilicity, and they have the ability to resist protein adsorption. PT and APTT are usually used to examine the extrinsic and intrinsic pathway of blood coagulation. When the coagulation time is longer, the fewer coagulation factors are activated. The high hydrophilic surface of modified PVDF membrane can significantly delay the activation of endogenous clotting system. Thus the modified PVDF has better intrinsic and extraneous antithrombogenicity than the virgin PVDF and Fresenius F60S membranes (Figure 7).

## 5. Conclusions

When PVP molecular weight is 30 KDa and the content is 3.0 wt %, the modified PVDF membrane has better mechanical and separation performance. The modified PVDF membrane by adding PVP has higher UF flux, LZM, and urea passage than virgin PVDF membrane. Compared with F60S hemodialysis membrane, the modified PVDF membrane has better mechanical and separation performance. It was proven that the modified PVDF membrane showed better antithrombogenicity, hydrophilicity, less BSA adsorption, and lower hemolytic ratio and adhesion of platelets.

## Figures and Tables

**Table 1:** Spinning parameters of hollow fiber membranes.

Spinning Conditions	Value
Bore fluid	DI water (70 V %) and DMAc(30 V %)
External coagulant	tap water
Bore flow rate (mL/min)	17.5
Dope flow rate (mL/min)	25
Length of air gap (cm)	2
Take-up speed (m/min)	56
Spinneret dimension (mm)	0.7/1.4

**Table 2:** Effect of PVP molecular weight on some performance of modified PVDF membranes, the modified PVDF membranes are M-a, M-b, and M-c with PVP molecular weights 6 KDa, 10 KDa, and 30 KDa, respectively.

Membrane Label	Porosity (%)	Bursting Pressure (MPa)	Max Pore Size (μm)
M-a	80.8	0.595	0.069
M-b	83.3	0.545	0.072
M-c	85.7	0.495	0.073

**Table 3:** Effect of PVP molecular weight on separation performance, the inner diameter and wall thicknesses of PVDF membranes were 200 and 40 µm, respectively. The length of membranes is about 20 cm. The surface area of membranes was 25 cm^2^.

Membrane Label	UF Flux (L·h^−1^·m^−2^)	Rejection of BSA (%)	Passage of LZM (%)	Passage of Urea (%)	Contact Angle (°)
M-a	483	93.2	52.5	93.2	49 ± 3
M-b	563	92.1	62.3	94.6	46 ± 2
M-c	684	90.8	72.6	95.4	38 ± 2

**Table 4:** Effect of PVP content on some performance of modified PVDF membranes, the modified PVDF membranes are M-1, M-3, and M-5 with PVP content 1.0%, 3.0%, and 5.0 wt %, respectively.

Membrane Label	Porosity (%)	Bursting Pressure (MPa)	Max Pore Size (μm)
M-0	76.2	0.625	0.067
M-1	84.1	0.565	0.071
M-3	85.7	0.495	0.073
M-5	90.4	0.370	0.087

**Table 5:** Effect of PVP content on separation performance, the inner diameter, and wall thicknesses of PVDF membranes were 200 and 40 µm, respectively. The length of membranes is about 20 cm. The surface area of membranes was 25 cm^2^.

Membrane Label	UF Flux L·h^−1^·m^−2^	Rejection of BSA (%)	Passage of LZM (%)	Passage of Urea (%)	Contact Angle (°)
M-0	313	91.3	53.2	94.4	52 ± 3
M-1	512	92.4	58.9	94.2	42 ± 2
M-3	684	90.8	72.6	95.4	38 ± 2
M-5	843	88.2	82.5	96.7	35 ± 2

**Table 6:** Some performance of PVDF and Fresenius F60S membranes, M-0, and M-c/M-3 are Virgin and modified Fresenius F60S membranes.

Membrane Label	Porosity (%)	Bursting Pressure (Mpa)	Max Pore Size (μm)
Virgin PVDF	76.2	0.625	0.067
Modified PVDF	85.7	0.495	0.073
Fresenius F60S	72.3	0.475	0.072

**Table 7:** Separation performance of PVDF and Fresenius F60S membranes, the inner diameter and wall thicknesses of membranes were 200 and 40 µm, respectively. The length of membranes is about 20 cm. The surface area of membranes was 25 cm^2^.

Membrane Label	UF Flux (L·h^−1^·m^−2^)	Rejection of BSA (%)	Passage of LZM (%)	Passage of Urea (%)
Virgin PVDF	313	91.3	53.2	94.4
Modified PVDF	684	90.8	72.6	95.4
Fresenius F60S	217	93.7	54.4	96.8

**Table 8:** Water contact angle and BSA adsorption of PVDF and Fresenius F60S membranes, the surface area of membranes was 100 cm^2^.

Membrane Label	Water Contact Angle (°)	BSA Adsorption (mg/m^2^)
Virgin PVDF	52 ± 3	110 ± 3
Modified PVDF	38 ± 2	45 ± 2
Fresenius F60S	64 ± 3	230 ± 4

**Table 9:** Hemolysis ratios of PVDF and Fresenius F60S membranes. Group 1, Group 2, and Group 3 were parallel experiments of standard direct contact method.

Membrane Label	Hemolysis Ratio		
	Group 1 (%)	Group 2 (%)	Group 3 (%)
Virgin PVDF	0.55 ± 0.13	0.63 ± 0.09	0.69 ± 0.08
Modified PVDF	0.27 ± 0.06	0.31 ± 0.05	0.26 ± 0.04
Fresenius F60S	0.74 ± 0.21	0.98 ± 0.25	0.76 ± 0.12
